# A HAND to TBX5 Explains the Link Between Thalidomide and Cardiac Diseases

**DOI:** 10.1038/s41598-017-01641-3

**Published:** 2017-05-03

**Authors:** Athar Khalil, Rachel Tanos, Nehmé El-Hachem, Mazen Kurban, Patrice Bouvagnet, Fadi Bitar, Georges Nemer

**Affiliations:** 1 Departments of Biochemistry and Molecular Genetics, American University of Beirut, Beirut, Lebanon; 2 Departments of Pediatrics and Adolescent Medicine, American University of Beirut, Beirut, Lebanon; 3 Departments of Dermatology, American University of Beirut, Beirut, Lebanon; 4 Department of Dermatology, Columbia University, New York, NY USA; 5 Laboratoire Cardiogénétique, INMG, CNRS UMR 5310, INSERM U1217 and Université Lyon 1, Lyon, France

## Abstract

Congenital heart disease is the leading cause of death in the first year of life. Mutations only in few genes have been linked to some cases of CHD. Thalidomide was used by pregnant women for morning sickness but was removed from the market because it caused severe malformations including CHDs. We used both in silico docking software, and *in vitro* molecular and biochemical methods to document a novel interaction involving Thalidomide, TBX5, and HAND2. Thalidomide binds readily to TBX5 through amino acids R81, R82, and K226 all implicated in DNA binding. It reduces TBX5 binding to DNA by 40%, and suppresses TBX5 mediated activation of the NPPA and VEGF promoters by 70%. We documented a novel interaction between TBX5 and HAND2, and showed that a p.G202V HAND2 variant associated with CHD and coronary artery diseases found in a large Lebanese family with high consanguinity, drastically inhibited this interaction by 90%. Similarly, thalidomide inhibited the TBX5/HAND2 physical interaction, and the in silico docking revealed that the same amino acids involved in the interaction of TBX5 with DNA are also involved in its binding to HAND2. Our results establish a HAND2/TBX5 pathway implicated in heart development and diseases.

## Introduction

Cardiac development is a key event in embryo formation in all organisms, mainly in vertebrates because it is the first organ to get fully functional ensuring the proper delivery of blood to all developing organs^1–5^. Macro and micro-environmental factors control all steps of cardiac chambers’ formation and function through a genetic network, highly conserved throughout species and organisms. The tight regulation of this process entails however a more severe damage to the whole embryo if any of the components of the machinery is derailed leading to congenital heart diseases (CHDs), which are still the leading cause of death in newborns worldwide^6–12^. At the DNA level, single nucleotide variants, small and large segment deletions, copy number variants (CNVs), and gross chromosomal structural abnormalities have been linked to different forms of CHDs. Yet, despite the exhaustive data generated from next generation sequencing (NGS), 80–90% of the cases are still with no known etiologies^6, 13, 14^.

In the pre-genetic/genomic era, many factors were thought to directly cause CHDs including the mother’s health conditions like age, pre-pregnancy BMI, gestational and type 1 diabetes, hypertension, and exposure to chemicals and teratogens. Among the chemicals were some prescribed medications like retinoic acid, Bupropion, and Thalidomide^15–17^. Thalidomide (Contergan®) was first introduced in 1956 as a sedative drug and was used in the treatment of nausea in pregnant women. Thalidomide, N-phthalimidoglutarimide [C13 O4 N2 H9], is a derivative of glutamic acid, and is pharmacologically classified as an immune-modulatory agent. It was removed from the market after causing severe birth defects in pregnant women taking it to relieve their “morning sickness”. The malformations include mainly congenital heart disease, and limb deformities in addition to malformations of the inner and outer ear, and ocular abnormalities. The exact mechanism by which Thalidomide triggers these malformations is still elusive, despite numerous publications on its effect on cell proliferation, DNA replication, transcription, synthesis and/or function of growth factors, synthesis and/or function of integrins, angiogenesis, chondrogenesis, and cell death^16, 18–20^. The only documented molecular target for Thalidomide is the cereblon protein (CRBN), a substrate receptor of the E3 ubiquitin ligase complex that plays a major role in targeted proteasomal degradation of proteins. It is through this interaction that involved also the Ikaros and Myc proteins, that the use of Thalidomide and its derivatives in treating myleomas and leprus (ENL) is now being understood opening thus the way to its FDA approved usage in treating different diseases^21–23^. This molecular target however doesn’t explain its effect on embryonic development especially at the level of the heart and limbs, since *CRBN* was not shown to play any major role in mice organogenesis^24^.

In the genetic era, mutations in *TBX5* were amongst the first to be linked to syndromic CHD cases by linkage analysis of patients with Holt-Oram syndrome (OMIM #142900). Holt-Oram syndrome (HOS) is a rare autosomal dominant disease (1 in 100 000) that causes mainly upper limb malformation and cardiac septal defects, similar to the ones observed in Thalidomide toxicity^25–28^. Mapped on 12q24.1, *TBX5* encodes a protein of 518 amino acids that possess a T-box domain that binds specifically to the DNA and interacts with other proteins in regulating the expression of different genes like *NPPA*, *GJA5*, *and OSR1* in the heart, and *FGF10* in the limbs^29–31^. *In vitro* and *in vivo* studies have shown that TBX5 is a strong transcriptional activator, and that its regulation occurs mainly via its interacting partners, GATA4,5,6, NKX2.5, and SALL4 and its cellular shuffling from the nucleus to the cytoplasm is via the CRM1 pathway^32–37^. In mice, the loss of one allele of *Tbx5* recapitulates the HOS phenotype, while the loss of both alleles lead to early embryonic lethality due to arrested cardiac cell growth, mainly atrial cells at E9.5^36, 38^. While Tbx5 is mainly expressed in the primary heart field in mice, and in particular in the myocardial cells, targeted inactivation of one copy of the gene in the endocardial cells lead to severe atrial septal defects identical to the ones observed in the general knock-out model^38^. With more than 40 reported mutations mainly affecting the T-box domain, there is no genotype-phenotype correlation despite *in vitro* characterization of the transcriptional properties of some of the mutated proteins. This has led to speculation about the post-transcriptional modifications of the transcripts and the identification of splice variants that could have different spatial and/or temporal pattern of expression.

While Tbx5 was shown to interact with cardiac-enriched transcription factors like the zinc finger GATA proteins, and the homeobox NKX2.5 protein, there are no reports about its interactions with members of the bHLH family, mainly the HAND1,2 and the HEY1,2,L proteins. Despite being expressed in a chamber-specific complimentary pattern in the heart, and despite playing major roles in heart formation in mice, the genes encoding these proteins were not associated so far to any form of CHDs.

We are hereby reporting the *in silico* and *in vitro* interaction between Thalidomide and TBX5, and the identification of a novel interaction between TBX5 and HAND2 targeted by the drug that could explain some of the embryonic malformations. Moreover, we are characterization a novel HAND2 single nucleotide variant (SNV) that is linked to a familial case of CHD, which leads to a mutated protein unable to physically interact with TBX5.

## Material and Methods

### Patient recruitment

The study was approved by the institutional review board (IRB) at the American University of Beirut (protocol number: Bioch.GN.01). All patients, and family members signed an informed consent form before being enrolled in the study. A total of 51 individuals from the same family were enrolled. Standard clinical evaluation included a complete physical exam, electrocardiography (ECG), and two-dimensional (2D) transthoracic echocardiography (TTE) with color Doppler was obtained. Family consanguinity history was utilized in constructing the pedigree after interviewing all patients and their parents. The study was conducted in adherence to the Declaration of Helsinki Principles.

### Genetic analysis

Peripheral venous blood was collected from each participant and genomic DNA was extracted from white blood cells using the Qiagen Blood-Midi kit (Qiagen Science Inc., Germantown, MD), as previously described^39^. Primers to amplify all coding exons were designed using the as per the genome.ucsc.edu PCR design. Amplification by polymerase chain reaction (PCR) was done using the Phusion polymerase high-fidelity master mix (F-548S) on a Pico machine (Finnzymes, Espo, Finland), and the amplicons were resolved on a 1.5% agarose gel. Gel purification was performed using the Gel Extraction kit following the manufacturer’s protocol (peqGOLD Gel Extraction Kit, PeqLab, Erlangen, Germany). The purified bands were quantified using a NanonDrop (Thermo Fisher Scientific Inc., Waltham, MA) and examined by gel electrophoresis to ensure quality. DNA sequencing was carried out on an ABI 3500 machine at the molecular core facility at the American University of Beirut, followed by analysis using the data collection software from Applied Biosystems Inc. (Foster City, CA).

### Cell Culture

Human embryonic kidney (HEK) 293 T cells, Hela cells, and JR1 rhabdomyosarcoma cells were maintained in DMEM culture media supplemented with 10% FBS and Penicillin streptomycin (P/S) and 1% Sodium pyruvate and incubated in a humid atmosphere 5% CO2 at 37 °C as previously described^39–41^.

### Immunofluorescence

Hela or JR1 cells were plated onto 12-well Costar culture plates on cover slips with 50,000 cells per well. Transient transfection was done on the second day of the seeding by polyethylenimine (PEI, Sigma) with a total of 4 μg of DNA per well. Cells were fixed 24 h post-transfection using 4% paraformaldehyde/PBS for 20 min and washed again with PBS. The cells were then blocked with 3% BSA/PBS solution for 1 hr followed by the primary antibody (rabbit anti-HA, sc-805, or mouse anti-Flag, sc-166355, Santa Cruz Biotechnology) diluted (1:200) in BSA/PBT and added to the cells with an overnight incubation at 4 °C. The cells were then washed with PBT 3 times, before adding the secondary antibody (donkey anti-rabbit IgG biotinylated, RPN1004V, or anti-mouse IgG biotinylated, GE Healthcare) with a dilution of 1:250 in BSA/PBT for 1 hour at RT with shaking. After washing 3 times with PBT, cells were incubated with Chromeo^TM^ 488 Streptavidin (Santa Cruz) for 1 hour at RT with shaking. Nuclei staining was also performed by applying Hoechst, diluted 1:30 in water, for 30 minutes. The cells were then washed with PBS and mounted on a rectangular slide containing an anti-fading agent DABCO (Sigma–Aldrich). The slides were examined using the Olympus BH-2 microscope at the molecular core facility in the faculty of medicine.

### Luciferase Assay

To analyze the transcriptional regulation of the mouse *VEGF* or rat *NPPA* promoters by TBX5, HAND2, and/or GATA4, HEK293 cells were plated in 12 wells culture plates with 50,000 cells/well. Transfections were done using 5 μl PEI/. Controls were transfected with either VEGF/Luc or *NPPA*/Luc and empty expression vector pCEP4 (Invitrogen), while the experimental wells were cotransfected also with the increasing doses of Tbx5. Plasmids harboring the full coding sequencing for the rat GATA4 gene, and mouse TBX5 or HAND2 gene were used as previously described^42^. After 24 hours 10 μl of thalidomide (20 mg/ml) and 10 μl of DMSO were added per well and incubated for 12 to 16 hours. Cells were then lysed with 150 μl/well 1X lysis buffer (1 mM Tris pH 8, NP40 10%) and left on the shaker for 20 minutes at room temperature. 100 μl of cell lysates was transferred into a 96 well plate (Costar) to which 100 μl of luciferin is added. Luciferin (Promega, Cat #E 1501) was prepared according to the manufacturer’s protocol. The signal was read immediately using the Fluoroskan Ascent (Thermo Fisher Scientific). The results were normalized to total protein concentration in each well, and were expressed as fold activation. The presented values are the mean ± standard deviation of three independent experiments carried out in duplicates.

### Western blot

For overexpression experiments, HEK 293 T cells were transfected using Polyethyleneimine^43^ with 20 μg of the plasmid encoding the murine Tbx5 isoform A. For Western blot analysis, the cells were harvested 48 hr after transfection, lysed, and nuclear proteins extraction was carried out as previously described. 100 mg and 200 mg of Thalidomide (T144, Sigma) dissolved in 5 and 10 μL DMSO respectively or DMSO (5 or 10 μL) were added to equal amounts of nuclear protein extracts (20 μg) of Tbx5 protein prior to loading on a SDS-gel. Western blot analysis using the primary the anti-HA antibody, and the secondary antibody conjugated with horseradish-peroxidase, anti-Rabbit-HRP was used to detect the Tbx5 protein Revelation was done using the Western Lightening Chemiluminescence Kit (GE Healthcare). The protein bands were visualized by autoradiography.

### Electrophoretic Mobility Shift Assay

In order to assess the binding affinity of Tbx5 on its consensus region in the presence of Thalidomide, electrophoretic mobility shift assay (EMSA) was done. The probe used corresponds to the T-half site of the rat *NPPA* TBE2 element as previously described and the HAND-consensus binding site “TCGACAGGGC**CATCTG**GCATTG” found also on the NPPA promoter^36^. The single strand primers were first annealed then phosphorylated with T4 polynucleotide kinase and γ-32P-ATP at the 5′-end. A radioactive labeled probe was obtained and migrated on a non-denaturing 12% Bis-Acrylamide gel for 45 minutes at 125 volts. The bands corresponding to a double stranded probe were cut accordingly and purified using Costar Spin-X columns (Costar, Cat #8161). The control binding reaction consisted of 10 μg of Tbx5 extract, 4 μl binding buffer (20 mM Tris pH 7.9, 120 mM KCl, 2 mM EDTA, 25 mM MgCl2 and 25% glycerol), 1 μl poly dI/dC (Amersham) and 1 μl of the probe. 5 or 10 μl of thalidomide (20 mg/ml) and 5 or 10 μl of DMSO were incubated on ice with the above mixture. The reaction was completed to 20 μl with water. After incubation for 20 minutes the samples were loaded on the 6% non-denaturing polyacrylamide gel and run for 2.5 hours in 0.25 X TBE buffer at 200 volts. BioRad gel dryer (Model 583) was used for 2 hours at 80 °C to dry the gel, which was then exposed to a phosphor imager screen. The screen is then scanned using the STORM (Molecular Dynamics) scanner.

### Co-immunoprecipitation Assay

After detecting Tbx5 and GATA4 proteins by western blot assay, co-immunoprecipitaion was done to assess the effect of Thalidomide on the interaction between Tbx5 (HA-tagged) and GATA4 (Flag-tagged). Protein A/G Dynabeads (Invitrogen) were captured on a magnetic stand and resuspended gently with PBS (1x + 0.001% of Tween 20). The beads were then incubated with rabbit anti-HA (Santa Cruz) for 1 hour at 4 °C on a rotating platform. 100 μg of Tbx5 is mixed with same amount of GATA4 and incubated with the antibody-complexed beads either alone, or with 200 mg thalidomide or 10 μl of DMSO. After 2 hours the mixture was washed 2 times with PBS (1x) and the pellets were resuspended in 2Х Laemmli buffer, boiled for 3 min, and run on a denaturing SDS-PAGE for 1.5 hr. Western blotting was performed with mouse anti- Flag (sc-166355, Santa Cruz), followed by anti-mouse IgG horseradish peroxidase (ab6789, Abcam) as previously described. Membranes were stripped using a stripping buffer (1 M Tris, SDS (10%), β- mercaptoethanol) for 30 minutes at 55 °C in a thermo-rotator followed by 3 times wash with TBT and blocked with 5% non-fatty milk for 45 min. The membranes were then incubated overnight with the rabbit anti-HA (Santa Cruz) antibody at 4 °C, washed 3 times with TBT and then incubated with the anti-rabbit IgG horseradish peroxidase-conjugated antibody (ab97051, Abcam) for 1 hour at room temperature. Another 3 consecutive washes were done before revelation that was done using the Western Lightening Chemiluminescence Kit (GE Healthcare). The protein bands were visualized by autoradiography.

### Chemicals

Thalidomide used in our project is from Sigma (T144). This drug is water insoluble, and was thus dissolved in DMSO. All the experiments were compared to DMSO to rule out any effect attributed to the solvent. The stock concentration of Thalidomide used in this study was 20 mg/ml.

### Docking

Protein-thalidomide docking simulations were performed using the default settings in Autodock-Vina^44^. Thalidomide 3D structure was docked to the T-box domain of TBX5 (pdbid: 2X6V) and TBX3 (pdbid: 1H6F) respectively, and the best docking poses were selected for further investigation. The program MODELLER^45^ was used to model the 3D structure of HAND2 bHLH domain based on the structure of the Scl:E47 bHLH Heterodimer bound to DNA (pdbid: 2YPB), and GATA4 DNA binding domain based on the crystal structure of GATA transcription factor-complex 3 (pdbid: 4HC9). Protein-protein docking simulations were submitted to the online webserver ClusPro (https://cluspro.bu.edu/login.php), a versatile protein-protein docking engine based on the FFT (Fast Fourier transformation). The engine outputs ranked models based on their corresponding cluster size and those with balanced coefficients were retained since there exist no prior knowledge of what forces prevail in the model^46^. Models from both protein-ligand and protein-protein docking were interactively visualized with Chimera (https://www.cgl.ucsf.edu/chimera/) and Discovery Studio (http://accelrys.com/).

### Statistics

Significance (*P < 0.05) for Luciferase, Gel Shift, and co-immuprecipitation assays was assessed using the one‐way ANOVA test. The likelihood of the p.G202V minor allele to be associated with congenital pathologies (e.g Tetralogy of Fallot) was assessed using family linkage analysis. We organized and analyzed our data using a parental transmission desiquilibrium test which incorporates phenotypes from both parents and offspring within and between nuclear families, a method initially described by Purcell *et al*., and applied in familiar based genetic association studies^47^.

## Results

Because of the similarities between the phenotypes in patients with HOS and those suffering from Thalidomide toxicity, we hypothesized that Thalidomide could directly bind to TBX5.

### The T-Box domain is the interface of the TBX5/Thalidomide interaction

**Figure 1:**
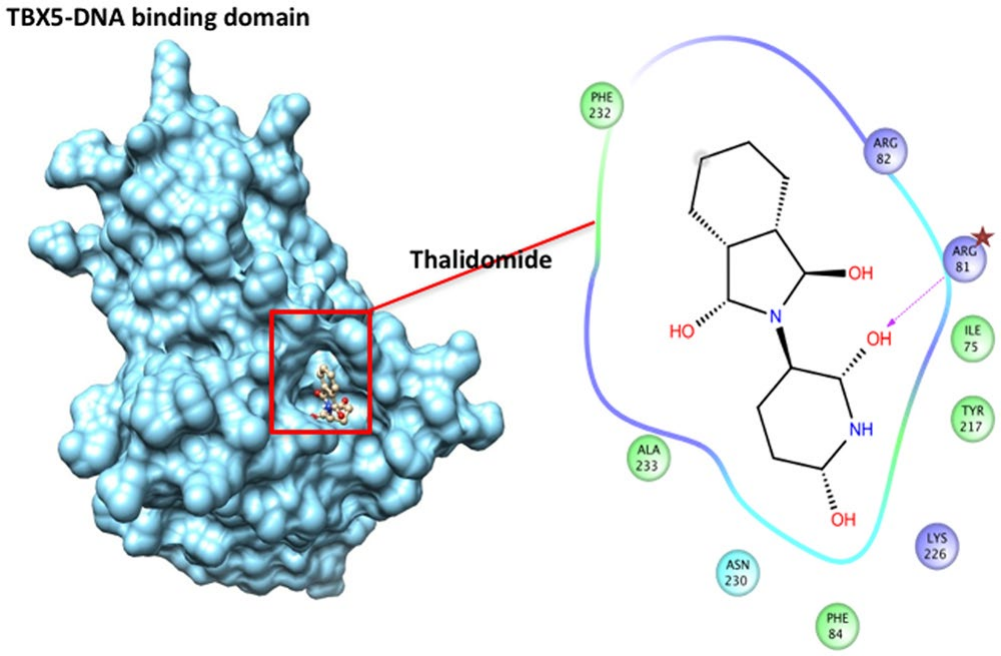
Thalidomide specific binding to the T-box domain of TBX5. The corresponding protein-ligand interaction diagram shows the most relevant amino acid within 5 Angstroms of thalidomide. Arginine at position 81 (arrow) is proposed as the main player for the TBX5-thalidomide docking simulation. The T-box domain of TBX5 (pdbid: 2X6V) was retrieved from the Protein Database (PDB). All colored amino acids with their positions are the ones in close proximity with Thalidomide.

Docking simulations using Autodock-Vina revealed a strong interaction between the T-box domain of TBX5 (pdb:2X6V) and Thalidomide. The top ranking pose was selected based on the best binding energy of ~7.5 kcal/mol (Fig. 1). Interestingly this interaction holds with TBX3 (pdbid:1H6F), a related member of the T-BOX family of transcription factors (Supplementary Figure 1). Although this interaction showed a similar energy pattern, the interacting residues do not involve those cooperating with DNA as in TBX5, which could suggest that the binding of thalidomide to TBX3 does not perturb its interaction with DNA. The Dissection of the interface region of TBX5 revealed a series of amino acids that make hydrogen bonds with the Thalidomide molecule, mainly R81 and 82, and K226 (Fig. 1). The same amino acids are also involved in the direct interaction of TBX5 to DNA.

**Figure 2:**
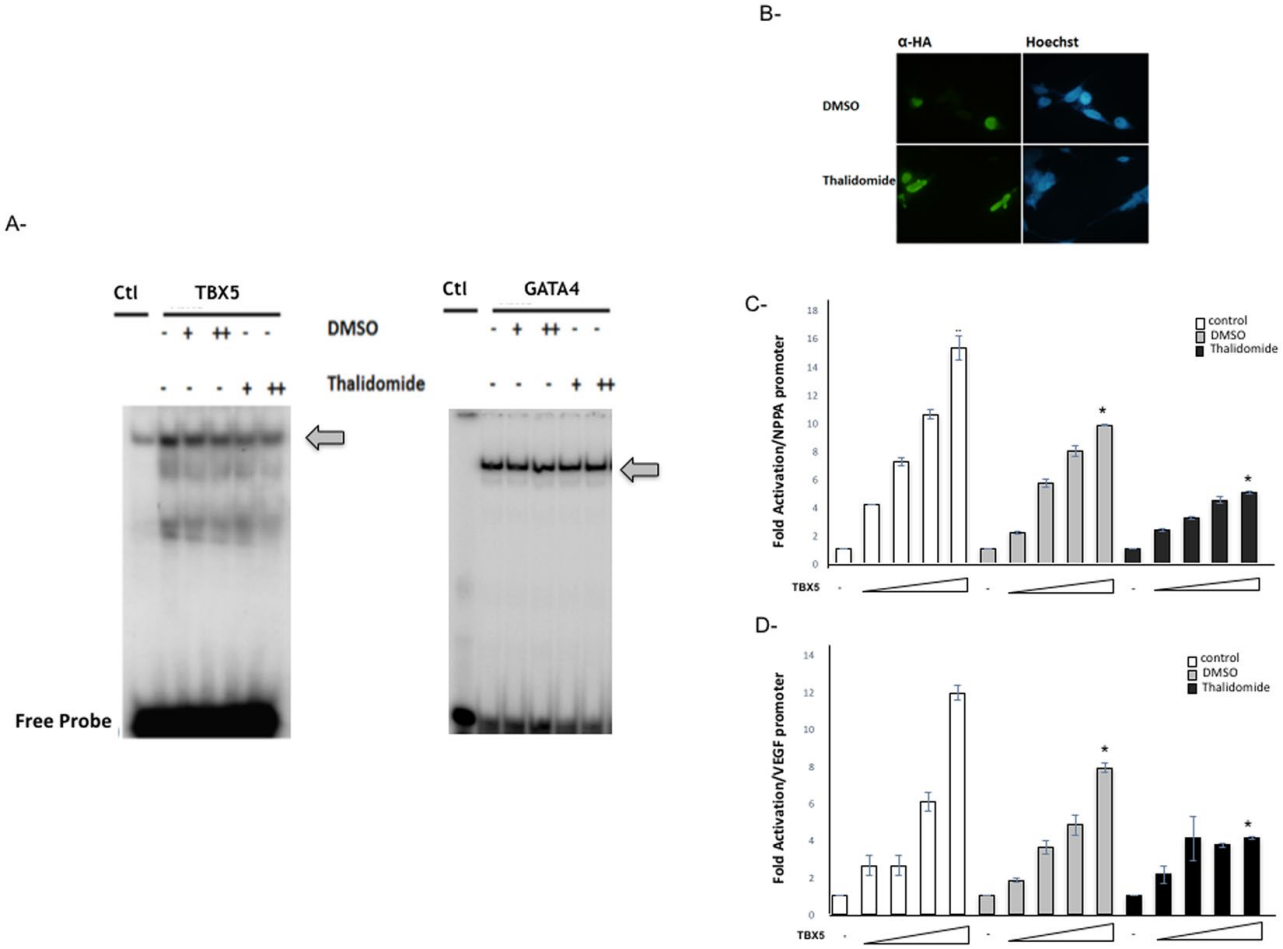
Thalidomide specific inhibition of TBX5 binding and activity. Interactions of TBX5 and GATA4 with the NPPA TBE and GATA sites was assessed by electrophoretic mobility shift assays using nuclear extracts from HEK293T cells overexpressing HA-TBX5 or Flag-GATA4 (**A**). The binding of TBX5 (arrow) is suppressed by 42% in the presence of 4 µM of thalidomide, while that of GATA4 (arrow) was not affected. (+2 µM, ++4 µM). DMSO treatment was used as a negative control. Immunofluorescence of JR1 cells transfected with the plasmid overexpressing a HA-tagged TBX5 protein and then treated with either 4 µM DMSO or thalidomide. The localization of TBX5 was visualized using an anti‐ HA antibody followed by a fluorescent secondary antibody (**B**). Nuclei of cells were visualized using the Hoechst dye (blue color). TBX5 showed nuclear localization (green color) in both conditions. (Magnification X40). Same results were obtained for Hela and Hek293 cells. Transcriptional activity was assessed by luciferase assay. Increasing doses of TBX5 (100 to 500 ng) were transiently co-transfected with the rat NPPA (**C**), or mouse VEGF (**D**) promoters coupled to luciferase in HEK293 cells. Treatment with thalidomide (4 µM) or its equivalent amount of DMSO was done after 24 hours, and cells were harvested for luciferase assay. Relative luciferase activities are represented as fold changes. The data are the means of 3 independent experiments done in duplicates ± SE. Significance (*P < 0.05) was assessed using the one‐way ANOVA test.

In order to confirm the *in silico* results, we carried out gel shift assays using the TBX5 protein overexpressed in HEK293 cells. Addition of increasing doses of Thalidomide (1 to 4 μM) to a mixture of 3 μg of nuclear extracts overexpressing TBX5 in the presence of a radiolabelled TBE (T-box element), leads to reduction in the binding complex by up to 40% with no change in protein stability (Fig. 2A, and Supplementary Figure 2). This inhibition is not observed using nuclear extracts overexpressing the GATA4 protein in the presence of its corresponding GATA probe. To test whether this inhibition occurs in the nucleus of living cells, we co-transfected the plasmid encoding TBX5 with its *bona fide* target the *NPPA* promoter fused to luciferase in HEK293 in the presence of 2 and 4 μM of Thalidomide and its equivalent amount of DMSO. We first assessed the effect of Thalidomide on the cellular localization of TBX5 by overexpressing the protein in different cell lines including, HK293, Hela, C2C12, and JR1. In all cases, and even at the highest dose of Thalidomide tested (4 μM), there was no change in the nuclear localization of the protein (Fig. 2B). Moreover, it didn’t lead to any proteolysis of the protein as assessed by Western Blotting. There was no toxic effect of Thalidomide on cell viability especially in HEK293 cells at the doses tested as assessed by MTT assays (Supplementary Figure 4). Transactivation of the *NPPA* and *VEGF* promoters were then assessed both in Hela and HEK293 cells, and the results showed that Thalidomide was able to inhibit TBX5-dependent activation reaching up to 70% at the highest dose (4 μM) (Fig. 2C,D). This comparison was done against the same dose of DMSO which reduces by up to 30% the transcriptional activity of TBX5.

### The TBX5-HAND2 pathway: a direct target for Thalidomide

**Figure 3:**
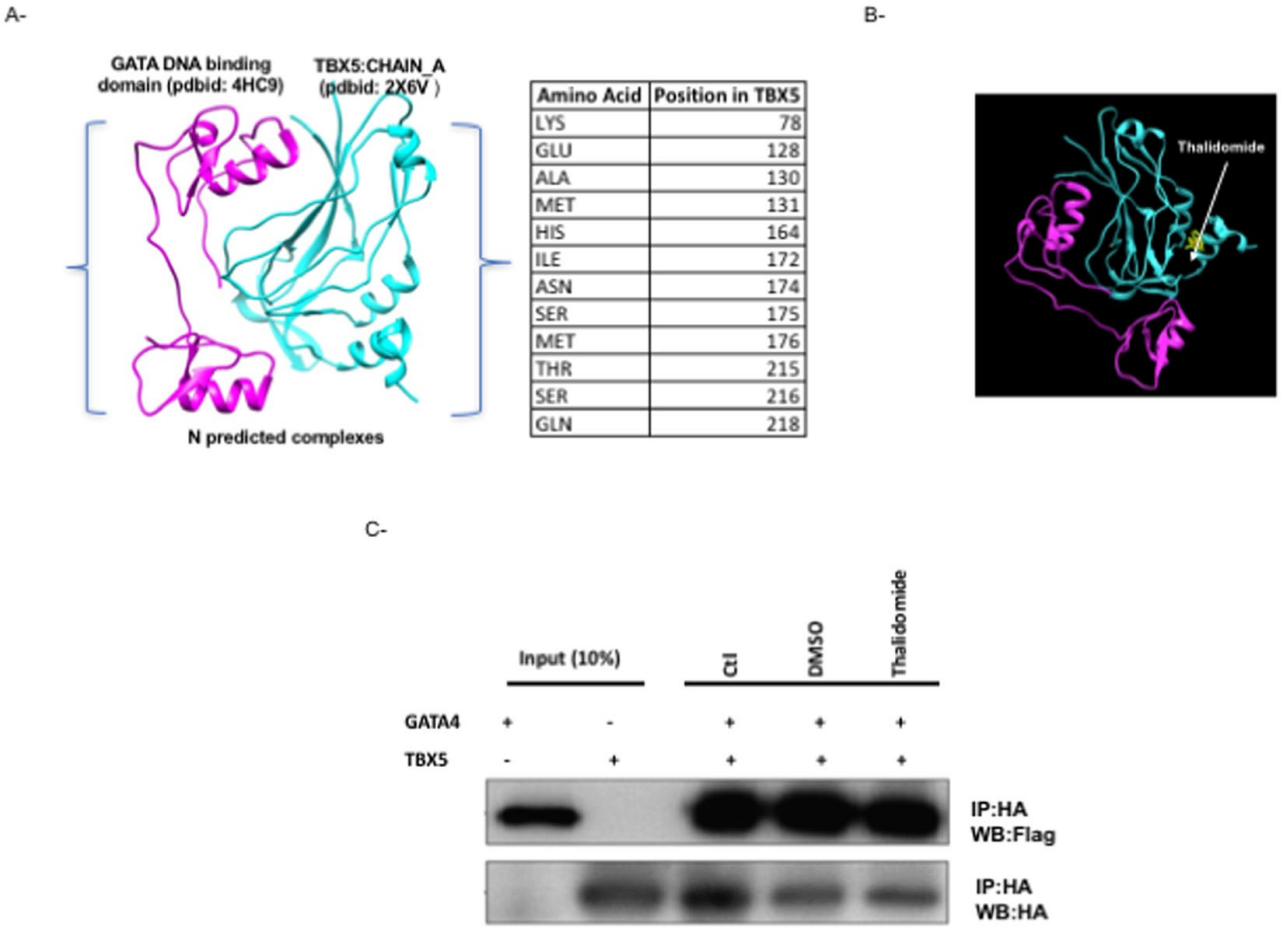
GATA4/TBX5 interaction is not affected by Thalidomide. Protein-protein docking interface between TBX5 and GATA4 modeled DNA binding domain as retrieved from PDB. Only Residues of TBX5 within 5 angstroms of this interaction are shown (**A**). The position of thalidomide in the context of the predicted TBX5-GATA4 interaction is clearly away from the interface region (**B**): TBX5 (cyan) and GATA4 (magenta). The physical interaction between both proteins was assessed by co-immunoprecipitation in the presence or absence (−) of either 4 µM thalidomide or DMSO (**C**). Ten times the quantity of proteins loaded for western blot was used for immune-precipitation. Nuclear lysates for both GATA4 and TBX5 were immune-precipitated with anti‐HA antibody and GATA4 proteins were visualized by western blot with an anti‐flag antibody. Membrane stripping and subsequent western blot analysis was performed with anti‐HA antibody in order to detect TBX5 proteins. Quantitation of the bands didn’t show any significant change. (Ctr: control, WB: Western-Blot, IP: Immuno-Precipitation).

**Figure 4:**
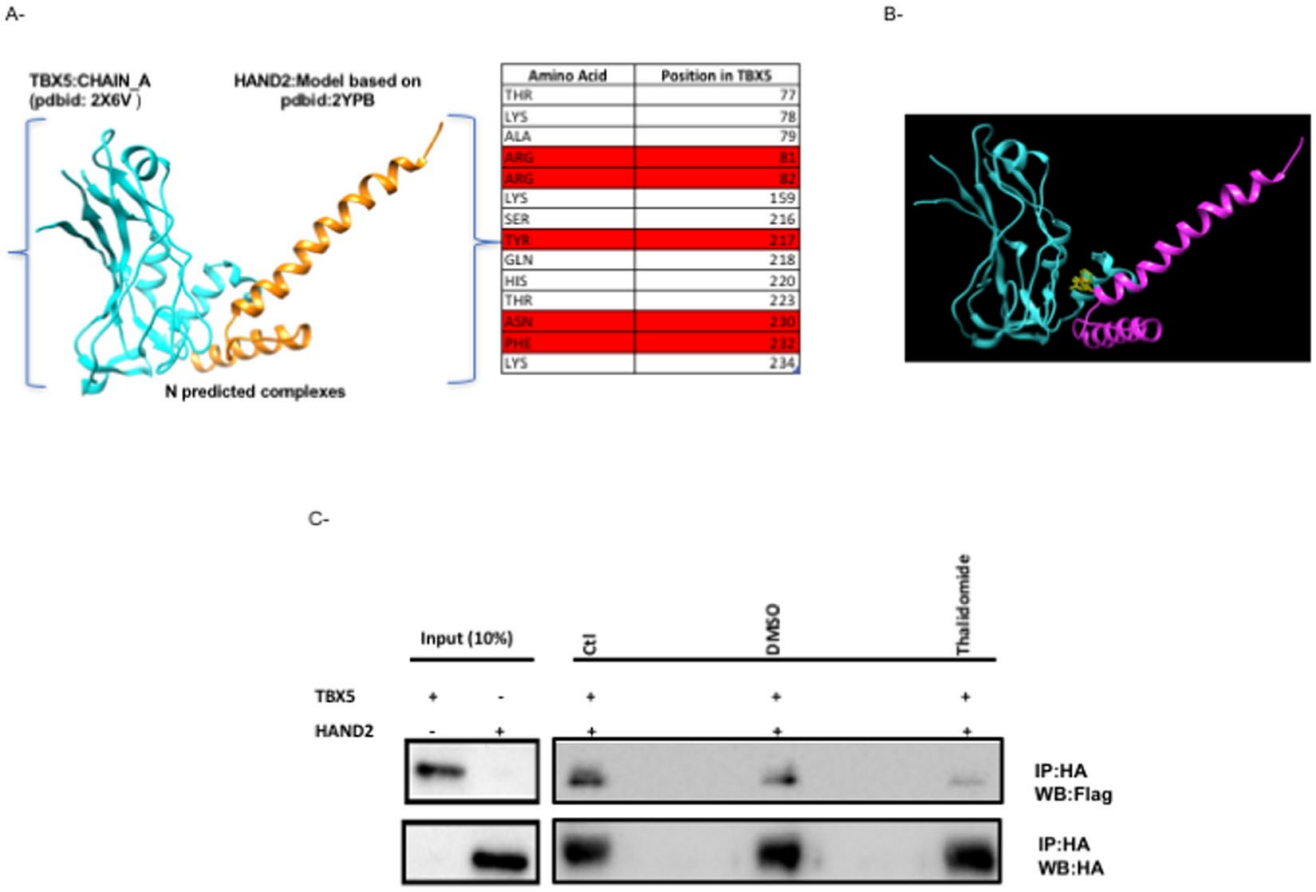
HAND2/TBX5 interaction drastically inhibited by Thalidomide. Protein-protein docking interface between TBX5 and HAND2 modeled DNA binding domain as retrieved from PDB. Only Residues of TBX5 within 5 angstroms of this interaction are shown (**A**). Those in red are associated with both TBX5-DNA and TBX5-HAND2 interaction from modeling simulation, which shows the position of thalidomide in the context of predicted TBX5-HAND2 interaction (**B**): TBX5 (cyan) and HAND2 (magenta). The physical interaction between both proteins was assessed by co-immunoprecipitation in the presence or absence (−) of either 4 µM thalidomide or DMSO (**C**). Ten times the quantity of proteins loaded for western blot was used for immunoprecipitation. Nuclear lysates of both HAND2 and TBX5 were immunoprecipitated with anti‐HA antibody and HAND2 proteins were visualized by western blot with and anti‐flag antibody. Membrane stripping and subsequent western blot analysis was performed with anti‐HA antibody in order to detect TBX5 proteins. Quantitation of the bands showed more than 80% decrease in the interaction when the extracts were incubated in the presence of Thalidomide. (Ctr: control, WB: immunoblot, IP: immnuoprecipitation).

TBX5 has been shown to interact readily with GATA4 and NKX2.5 during heart development^48, 49^. We used the ClusPro docking algorithm to predict protein-protein interactions between GATA4 and TBX5 in order to map the amino acid interface. Since we have no clues about the type of the forces (electrostatic or hydrophobic) that drive the interaction, we only select those with balanced energy profiles using the available T-box domain (pdbid: 2X6V), and GATA zinc finger carboxy-domain (pdbid: 4HC9). Models with lowest score were taken into consideration and inspected visually. As shown in Fig. 3, many amino acid residues in TBX5 are within 5 Å from GATA4, and these amino acids differ from the ones used by TBX5 to bind DNA and interact with Thalidomide. We used the previous TBX5 structure bound to Thalidomide, and dock it to GATA4. The binding of Thalidomide to TBX5 didn’t pose any hindrance to the TBX5/GATA4 interface suggesting that *in vivo* this interaction is preserved (Fig. 3A,B). We therefore tested the physical and functional interaction between the two proteins in the presence of Thalidomide, and showed that even at higher concentrations, Thalidomide was not able to suppress the intermolecular interaction between the two proteins (Fig. 3C). These results prompted us to look at potential partners for TBX5 present both in the limbs and in the heart that could be targeted by Thalidomide. We therefore used the same modeling strategy as for GATA4 to characterize the amino acids in TBX5 that directly interact with HAND2 by using the bHLH domain of Pan/E47 (pdbid: 2YPB) as a model. The results showed that in sharp contrast to GATA4, 5 out of the 14 amino acids – R81, R82, Y217, D230, and F232- involved in this interaction are common for the interaction with Thalidomide (Fig. 4A,B). We therefore assessed the effect of Thalidomide on the physical interaction between the two proteins, and showed that it was drastically inhibited by the drug (up to 80%) (Fig. 4C).

### HAND2: a novel marker of CHD

**Figure 5:**
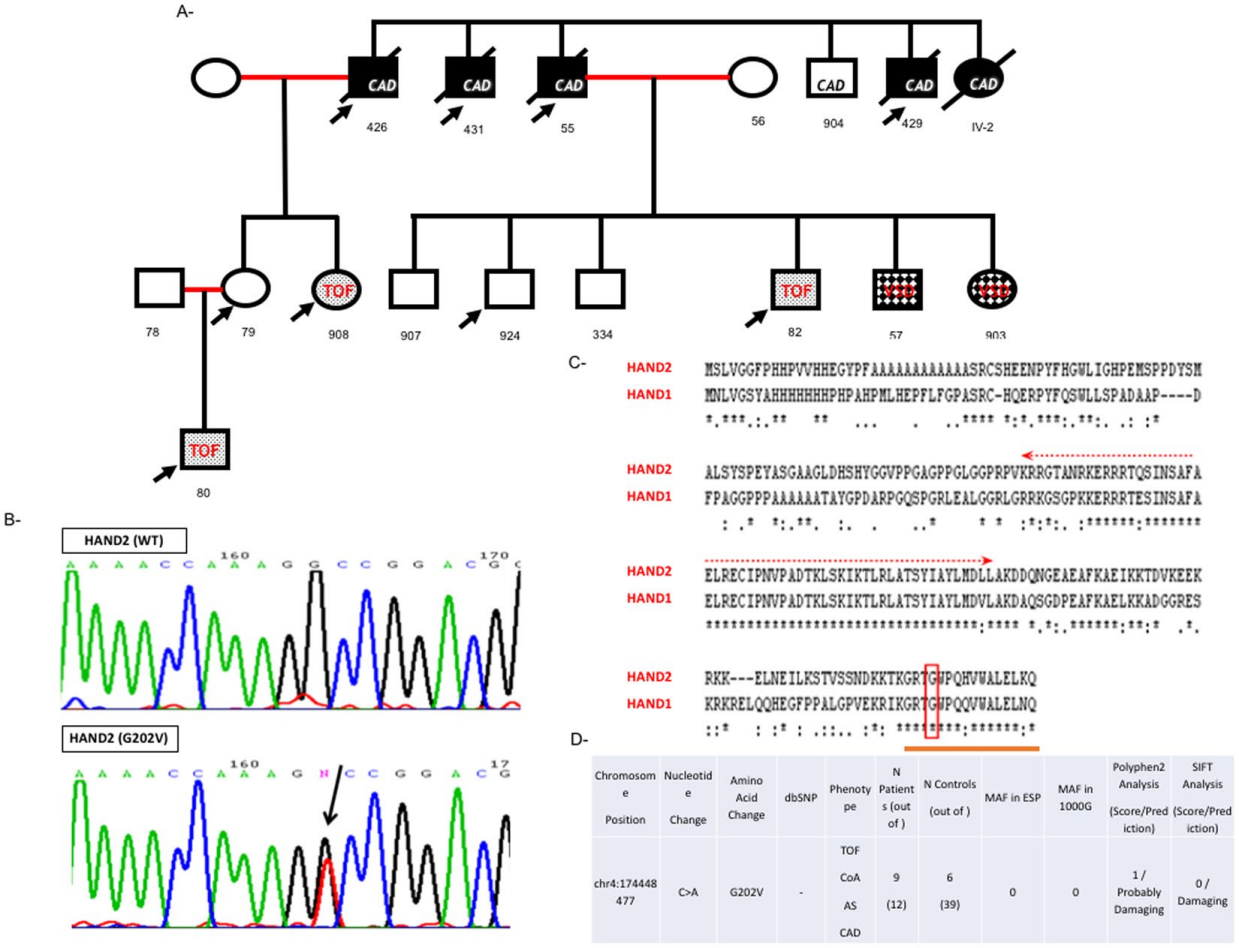
Phenotype/Genotype of the p.G202V HAND2 within the affected family. The pedigree (**A**) was annotated using the clear squares and circle for healthy males and females in the family. The marriages between first-degree cousins were annotated as red lines, and the affected individuals with the appropriate symbols. The p.G202V variant was indicated by arrow on all affected individuals. All deceased individuals were annotated with black strikethrough squares or circles. Numbers underneath each refer to the code given for each DNA sample corresponding to the individual. (CAD: coronary artery disease, TOF: tetralogy of Fallot, VSD: ventricular septal defect). Chromatograms (**B**) showing Sanger sequencing results for WT and the p.G202V variant. The boxed region corresponds to the heterozygous C > A nucleotide change read with the reverse primer. Sequence conservation of the G202 amino acid at position 202 between the HAND proteins (HAND1 and 2) using the clustalw tool (www.ebi.ac.uk/Tools/msa/clustalw2/). The Glycine residue is boxed within the highly conserved C-terminal domain while the conserved bHLH domain is marked with an arrow (**C**). The genotype and phenotype of the extended family members are listed along the minor allele frequency (MAF) and the in silico predictions of the mutation (**D**).

In order to link the above findings to the causes of CHDs, we screened 5 families with different forms of cardiac malformations for mutations encoding the main classes of transcription factors in the heart using Sanger sequencing. We were only able to find one novel variant in HAND2 in one family, while the other 4 families didn’t show any potential variant in genes encoding GATA4,5,6, NFATC1, TBX1,2,3,5,20, HAND1, and Hey1, 2 (Fig. 5, and data not shown). The chr14:174448477C > A variant which leads to a missense variant at position 202 of the protein, was only found in 5 out of 7 patients with different forms of Congenital Heart Disease, three of them with Tetralogy of Fallot (TOF), and the other two with Coarctation of the Aorta (CoA) or Aortic Stenosis (AS) (Fig. 5, and Supplementary Figure 3). The small indexed family (Fig. 5) was part of a large family with many consanguineous marriages and multiple CHDs and coronary artery diseases (CAD) (Supplementary Figure 2). We were able to recruit 51 individuals from this family and we genotyped all of them for this variant. In addition to the cases mentioned previously, the variant was observed in 4 patients who suffered early severe myocardial infarct that lead to death in their mid-40’s (Supplementary Figure 3). It was also found in 6 out of 39 healthy individuals within the extended members of this family (Supplementary Table 1). This variant was however, neither found in healthy individuals in the ExAC exome nor in the 1000 genome databases, nor in our cohort of 300 patients with CHDs and 500 healthy controls, suggesting it could have a deleterious effect on protein function (Fig. 5D). Furthermore, the asymptotic p-value from the parental transmission desiquilibrium test is 0.045 for the studied family. This provides a promising rationale that the p.G202V variant is linked to cardiac congenital conditions.

**Figure 6:**
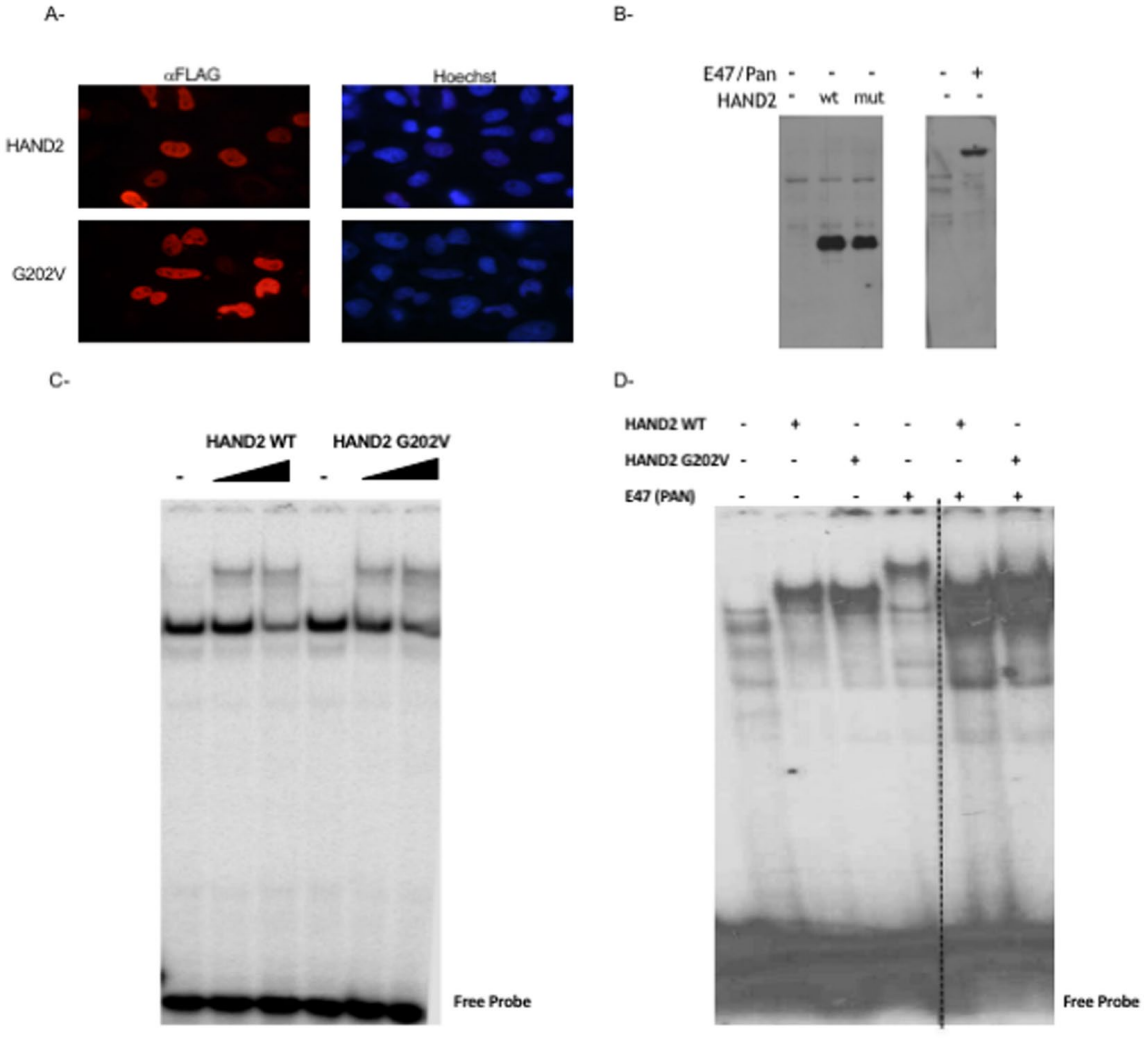
Characterization of the p.G202V protein *in vitro*. Transiently transfected Hela cells with plasmids harboring either the wild type HAND2 or the p.G202V variant were fixed and stained with an anti-Flag antibody. The secondary fluorescent antibody showed no change in the nuclear staining (in red) for both proteins as compared to the Hoechst staining (Magnification X40) (**A**). Western blot using the same anti-Flag antibody shows equal expression of both the wild type (wt) and mutated (mut) proteins in Hek293 cells, as well as the transiently overexpressed E47/Pan protein (**B**). Gel Shift analyses with increasing doses from the same nuclear extracts as in (**C**) were used on a consensus HAND binding site showed similar pattern of binding for both the wild type and mutant HAND2 proteins (**D**). The same pattern was observed when mixing the same amount of the HAND2 proteins with E47/Pan. The panel was separated by dotted lines to highlight the fact that the presented figure is a merge of two different gels run simultaneously.

**Figure 7:**
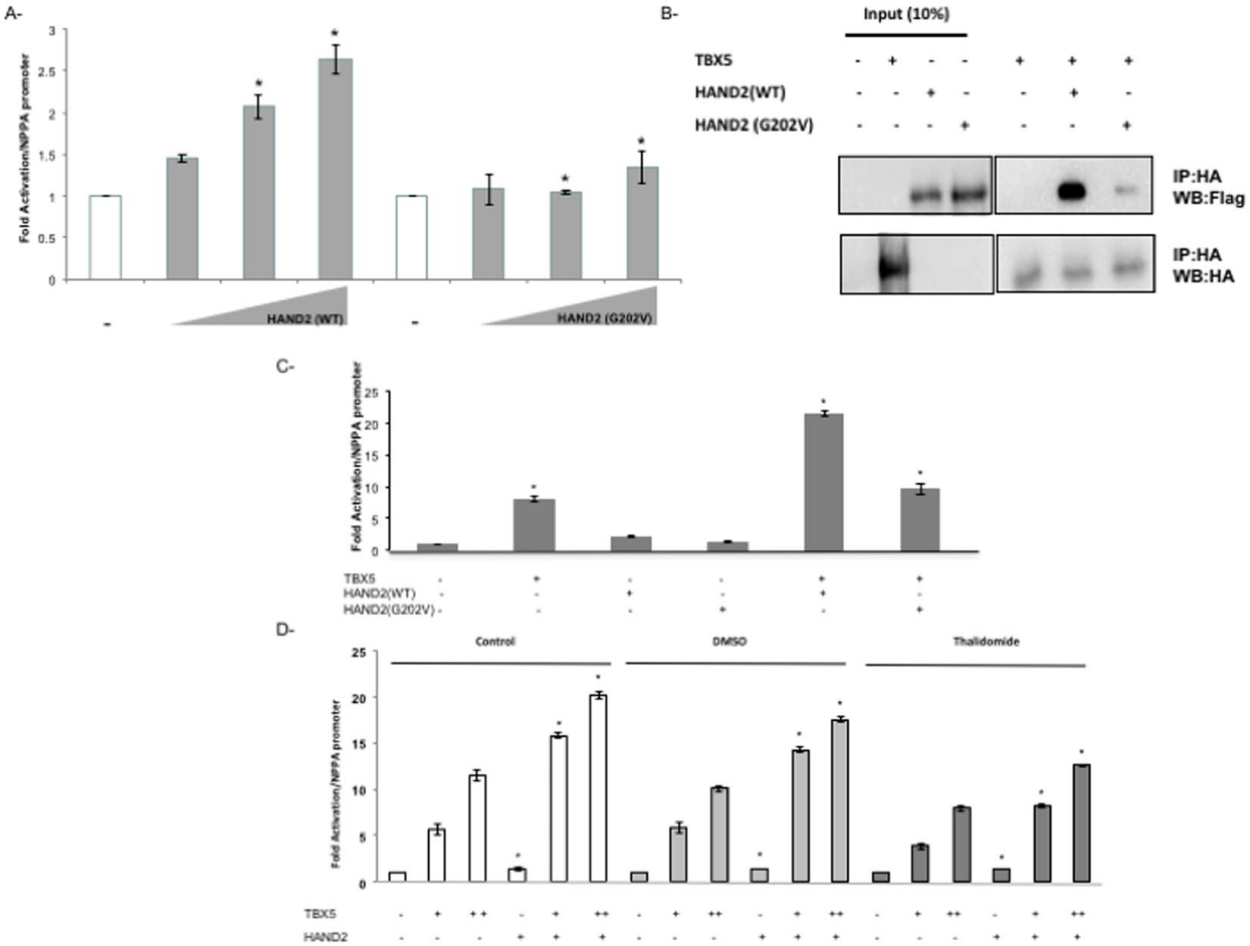
The TBX5/HAND2 Functional Interaction Inhibited both by the p.G202V variant and Thalidomide. The transcriptional activity of HAND2 was assessed in co-transfection assays using the rat NPPA promoter coupled to luciferase which showed a drastic decrease in the p.G202V mutant ability to activate its target (**A**). Relative luciferase activities are represented as fold changes. The data are the means of 3 independent experiments done in duplicates ± SE. Significance (*P < 0.05) was assessed using the one‐way ANOVA test. HA‐tagged TBX5 and Flag‐tagged HAND2 (WT and G202V) proteins were extracted from transiently transfected HEK293 cells. Nuclear lysates for both HAND2 and TBX5 proteins were immune-precipitated with anti‐HA antibody, and visualized with western blot via anti‐flag antibody. Membrane stripping and subsequent western blot analysis was performed with anti‐HA antibody in order to detect TBX5 proteins. Ten times the quantity of proteins loaded for western blot was used for immune-precipitation. Quantitation of the bands shows up to 80% inhibition of the interaction when the HAND2 mutated protein is used instead of the wild type protein (**B**). Transcriptional activity was assessed by luciferase assay (**C**,**D**). TBX5 and HAND2 (WT or G202V) were transiently co-transfected with the rat NPPA promoter coupled to luciferase in HEK293 cells (**C**), then in the presence of Thalidomide (**D**). Relative luciferase activities are represented as fold changes. The data are the means of 3 independent experiments done in duplicates ± SE. Significance (*P < 0.05) was assessed using the one‐way ANOVA test. (+) is for 300 ng of DNA, and (++) for 700 ng of DNA. The increasing doses start at 100 reaching up to 500 ng.

We then used two different bioinformatics tools - Polyphen2, and SIFT- to carry on *in silico* analysis of the amino acid variation effect on HAND2 structure and function. Both predict a strong damaging effect for the p.G202V variant on the protein (Fig. 5D). Taken altogether, and given the fact that the Glycine residue is evolutionary conserved amongst HAND2 proteins and even between HAND1 and HAND2 (Fig. 5C), we generated the plasmid carrying the missense variant by site directed mutagenesis and assessed its transcriptional properties as compared to the wild type protein. Our *in vitro* characterization of the protein showed that this variant didn’t affect cellular localization of the protein, nor its DNA binding properties as assessed by gel shift analysis with or without its partner Pan (Fig. 6A–C). Functionally, the p.G202V mutations led to a 50% decrease in the transcriptional activation of HAND2 over the *NPPA* promoter as assessed by Luciferase assays (Fig. 7A).

We then assessed whether the mutation affects the physical interaction between HAND2 and TBX5. Co-immunoprecipitation assays in Hek 293 cells showed that the p.G202V alteration inhibited by up to 90% this interaction (Fig. 7B). In addition, we establish a functional interaction between the two proteins over the NPPA promoter, a synergy that was totally abolished by the p.G202V mutation (Fig. 7C). In fact, TBX5 and HAND2 were able to synergistically activate the *NPPA* promoter by up to 22 fold, and this synergy was completely lost with the mutation, while being slightly affected by DMSO (Fig. 7C). Interestingly Thalidomide did also inhibit this synergy the same way the p.G202V did it with no effect on the transcriptional activity of HAND2 alone on the NPPA promoter (Fig. 7D).

## Discussion

Thalidomide toxicity and tight regulation of heart development and cardiac morphogenesis were tightly linked albeit the absence of a key-lock system. We provided in this manuscript as the first evidence that TBX5 is a direct target for Thalidomide explaining thus the cardiac phenotype and potentially the limb deformities in children whose mothers were exposed to this drug. In addition, we characterize a novel pathway that engages both transcription factors TBX5 and HAND2 in a common pathway in heart and limb development and in Thalidomide toxicity.

### TBX5/Thalidomide: the T-box pocket

The importance of identifying a specific target for Thalidomide that would explain its tissue and organ specific teratogenicity is of crucial importance in light of the recent usage of this drug in cancer treatment. The dilemma that has faced most researchers in the field about Thalidomide toxicity is the differential effect it had on rodent embryos (rats, and mice) versus human embryos. This was attributed mainly to the differential half-life of Thalidomide in blood plasma, and the high reservoir of anti-oxidants in mice and rats versus humans. Despite the documented thirty or so mechanisms of action which include cell death and DNA synthesis, none could explain all the damages caused in the embryo. We have shown in this work that Thalidomide binds directly to TBX5 on R81, R82, and K226, the same amino acids implicated in binding to DNA. Interestingly these amino acids are highly conserved in members of the T-box of family of transcription factors raising the possibilities that this interaction might be T-box specific. The specific effect on TBX5 could at least explain the phenotypes observed in children who suffered from Thalidomide toxicity at the level of the heart and limbs whereby the phenotypes are strikingly similar to patients with HOS harboring mutations in *TBX5*. The fact that the in silico docking data showed less affinities for Thalidomide to TBX3 versus TBX5, though both share the same docking interface to DNA^50^, argues for a differential effect of Thalidomide on various T-box proteins which would explain the tissue-specific pattern observed in children who suffered from its effect on their mothers. In the heart, the similarity between Holt-Oram patients and Thalidomide embryopathy argues for a specific interaction with TBX5 and not with other members of the T-box family normally expressed during cardiac development. Elsewhere in the embryo, the expression of both TBX 4, and 5 in the limbs for example can provide the T-box pocket for Thalidomide’s action. In other organs like the skin, where TBX3 is expressed, no toxicity was observed in concordance with our predicted low binding affinity to Thalidomide. Previously, only one protein has been reported to directly interact with Thalidomide: Cerbelon (CRBN)^23, 51–53^. However, despite its role in ubiquitination and its enriched expression in the brain, the inactivation of the *CRBN* gene in mice didn’t lead to any embryopathy that resembles Thalidome toxicity, but rather led to a mild learning disability that was also observed in humans with a mild type of autosomal recessive non-syndromic mental retardation due to loss of function *CRBN* mutations^24, 54, 55^. Our results provide thus a more plausible direct mechanism by which Thlaidomide would cause defect in organogenesis. One potential downstream target of this interaction could be the growth factor VEGF involved in angiogenesis and vasculogenesis, which we showed is a direct target of TBX5 and is significantly inhibited *in vitro* by Thalidomide (Fig. 2). The importance of this protein in relaying the observed phenotype came from recent studies, which showed that phocomelia, the major phenotype in Thalidomide embryopathy is linked to defects in angiogenesis precisely via VEGF^53, 56, 57^. Whether the interaction happens in the cytoplasm or in the nucleus is another challenging question to answer. It is well established that Thalidomide as a small molecule or its metabolites can cross the cell membrane, but it is not known whether it can enter the nucleus^16, 58^. Although our results didn’t show any effect of Thalidomide on TBX5 cellular localization, the fact that TBX5 is highly regulated by posttranscriptional mechanisms generating many isoforms, as well by post-translational mechanisms through its nuclear export signaling makes it thus the ideal protein target for Thalidomide^59^. Adding to this unique signature is the highly selective panel of interacting proteins with which TBX5 is associated, and to which we added in the current work the bHLH protein HAND2^33, 36, 59–62^.

### HAND2/TBX5: A novel bHLH/T-Box pathway in cardiovascular development and disease

The Thalidomide embryopathy has contributed largely to increasing awareness about the genetic/environmental axis as a playground for more than one player, hence the shift lately towards exome screening of patients with CHDs rather than the conservative candidate gene approach. The technology was not however available when we started screening large families with more than 2 patients with CHDs at the American University of Beirut. Our screen for such families using the candidate gene approach was not successful except for a couple of families including the one discussed hereby. The p.G202V variant in *HAND2* is the first variant that affects a conserved region in the C-terminal part of the protein leading to a transcriptionally inactive protein. Besides four reports, two of them published recently, there is no genetic data linking *HAND2* variants to congenital diseases^63–66^. Our results suggest a strong association of the p.G202V variant with the TOF and aortic malformations phenotypes, but not with the VSD phenotype (Supplementary Table 1 and Fig. 5). This correlates with the expression of HAND2 during cardiac development, which is highly restricted to the Outflow Tract and right ventricle^67–74^. We ruled out the involvement of known CHD players in patients within this family, by targeted next generation sequencing of 119 of such genes as previously described^75^. This should however be further evaluated by increasing the number of affected patients/families since the parental transmission disequilibrium test shows a moderately conservative p-value. Whereas the recent article by Lu CX *et al*. does suggest a direct link between the p.L47P *HAND2* variant and TOF^66^, we suggest that our p.G202V variant doesn’t contribute to all four phenotypes observed in Tetralogy of Fallot, but rather contribute to at least one of them probably the one pertaining to the overriding aorta, since the same variant is also found in both patients with CoA and aortic stenosis (Supplementary Table 1). In addition, the presence of the variants in a couple of healthy individuals argues for a partial penetrance pattern of inheritance, although some of those individuals are young and may suffer from aortic stenosis later on or even succumb to early myocardial infarcts. The aorta and outflow tract are in fact mostly affected in the knock-out *Hand2* mouse model whereby the *Hand2* −/− embryos exhibit hypoplasia of the right ventricle, branchial arches, and aortic arch arteries, one again backing our hypothesis that a defect in one allele of HAND2 might only contribute to a particular phenotype within the spectrum of phenotypes observed in patients with TOF^68, 71, 73, 74^. In parallel, an additional phenotype that could particularly be associated with the p.G202V variant is the coronary artery disease (CAD) phenotype that caused early death of individuals in their mid-40s because of a severe myocardial infarct. The fact that the p.G202V variant was present in all 4 patients who succumbed to MI suggests a role for this variant in coronary arteries. The still 6 healthy individuals carrying the variant are less than 40 years of age, and thus should be monitored to prevent any myocardial infarct. This is corroborated by the recent finding in mice, whereby Hand2 is a downstream effector of endocardial NOTCH signaling, in regulating both cardiogenesis and coronary vasculogenesis^76^. In fact, mice lacking endocardial *Hand2* have an increased density of coronary lumens and a dis-regulated VEGF signaling signature. Importantly, we showed that the mechanisms by which this mutation is affecting HAND2 function is by interfering with its transcriptional activity without affecting neither its binding affinity to DNA, nor its cellular localization. HAND proteins are however not very potent transcriptional activators, but rather contribute to the activation of their effectors through combinatorial interaction with other proteins. In our case, the *bona fide* partner for HAND2, the E47 protein was not affected by the mutation, and both proteins were still able to form highly stable heterodimers (Fig. 6) despite losing their synergy over their NPPA target promoter (data not shown). This was also the case with GATA4 and NKX2.5 which still were able to synergistically activate the NPPA promoter with the p.G202V HAND2 protein (data not shown). Only TBX5 was no more able to interact both physically and functionally with the HAND2 mutated protein highlighting the specific effect of the mutation on its function. This interaction, the first between a T-box protein and the HAND bHLH subfamily would only have a valuable significance, in the context of the observed phenotypes in the affected family. In the context of the TOF phenotype and the aortic stenosis/coarctation phenotypes, we hypothesize that the HAND2 mutation is solely linked to the phenotype because TBX5 and HAND2 expression is not overlapping in any of the 4 affected structures (ventricular septum, aorta, right ventricle, and outflow tract). In contrast, and of particular interest is the coronary artery disease problem which could be explained by the defective interaction of the p.G202V HAND2 protein with TBX5. In fact, both TBX5 and HAND2 are expressed in the pro-epicardial cells and play important roles in the formation and maturation of the coronary arteries^76–79^. Note that our results do suggest a more prominent role for this interaction in both congenital heart disease and coronary diseases, but the fact that only one copy of the *HAND2* gene is affected in this particular family makes it difficult to prove our hypothesis. However, the fact that Thalidomide drastically inhibits the interaction of HAND2 with TBX5 but not that of TBX5 and GATA4 opens the way for a better understanding of the mechanism by which TBX5/HAND2 could control cardiac and limb development in particular in regions where they are both co-expressed, and where they both could regulate target downstream genes like VEGF.

## Conclusion

Our study describes the first direct target for Thalidomide that could explain its embryopathy based on similarity to the HOS phenotype. It highlights the importance of a novel pathway in heart development and cardiac diseases that involves bHLH and T-box proteins. This opens the way to a better understanding of such interactions during embryogenesis and in pathological conditions where members of both families of proteins are co-expressed. Of particular interest, the recommendation to screen for HAND2 and TBX5 variants in families at risk for early and fatal myocardial infarcts.

## Electronic supplementary material

Supplementary Info
