# Supplementary material for: A HAND to TBX5 Explains the Link Between Thalidomide and Cardiac Diseases
Source: Sci Rep. 2017 May 3;7:1416. doi: 10.1038/s41598-017-01641-3 (PMC5431093; doi:10.1038/s41598-017-01641-3)
Supplement: Supplementary file 1 — Supplementary Info [file 41598_2017_1641_MOESM1_ESM.pdf]

A HAND to TBX5 Explains the Link Between Thalidomide and Cardiac Diseases

Running Title: The HAND2 and TBX5 Pathway in the Heart

Athar Khalil[1], Rachel Tanos[1], Nehmé El-Hachem[1,6], Mazen Kurban[1,3,4], Patrice Bouvagnet[5], Fadi Bitar[1,2], and Georges Nemer[1,*]

Departments of [1]Biochemistry and Molecular Genetics, [2]Pediatrics and Adolescent Medicine, and [3]Dermatology, American University of Beirut, Beirut, Lebanon

[4]Department of Dermatology, Columbia University, New York, NY, USA

[5]Laboratoire Cardiogénétique, INMG, CNRS UMR 5310, INSERM U1217 and Université Lyon 1, Lyon, France

[6] Integrative systems biology/bioinformatics, IRCM, Montreal, Montreal, Canada

Supplementary Table 1: Phenotype/Genotype of the extended family members for the p.G202V HAND2 variant

Supplementary Figure 1: Differential interaction of Thalidomide with TBX3 versus TBX5
The corresponding protein-ligand interaction diagram shows the most relevant amino acid within 5 Angstroms of thalidomide. Note the different amino acids between TBX3 and TBX5 that get in close proximity to thalidomide. All amino acids that are implicated in the interaction with Thalidomide are listed in yellow.

Supplementary Figure 2: Expression of TBX5 proteins used in EMSA
15 micrograms of nuclear extracts from HEK293 cells overexpressing HA-tagged TBX5 were resolved on a denaturating gel under different conditions (control, DMSO, and Thalidomide). Western blot with HA ploycloncal rabbit antibody shows equal amount of proteins under all conditions.

Supplementary Figure 3: MTT assay on HEK293 cells treated with Thalidomide
The data are represented of %cell survival over 24 hours after treatment with Thalidomide (4µM) or DMSO. The numbers represent the mean ± Standard deviation for 3 experiments done in triplicate.

Supplementary Figure 4: Genetic analysis of the p.G202V variant within the large affected family
The pedigree includes all individuals over 6 generations (roman numbers). All coded DNA samples numbers (arabic) are underneath each corresponding individual. Those individuals whom we have a "hear-say" account from family members are given roman numbers. The rest are not numbered.

Supplementary Table 1: Phenotype/Genotype of the extended family members for the p.G202V HAND2 variant

Patient ID	Phenotype	HAND2-p.G202V
78		-
79		+
80	TOF	+
81		-
82	TOF	+
890		-
891		-
892		-
894		-
895		-
896		-
897		-
898		-
899		-
900		+
901		-
902		-
903	VSD	-
904	CAD	-
905		-
906		+
907		-
908	TOF like + PA	+
909		-
910		-
911		-
912		-
913		-
914		-
915		-
916		-
917		-
918		+
919		+
920		-
921		-
922		-
923		-
335		+
925		-
926		-
227	AS	+
228		-
229	CoA	+
427		-
429	CAD	+
334		-
55	CAD	+
426	CAD	+
431	CAD	+
57	PDA + VSD	-

TOF: Tetralogy of Fallot
VSD: Ventricular Septal Defect
CAD: Coronary Artery Disease
PA: Pulmonary Atresia
AS: Aortic Stenosis
CoA: Coarcation of the Aorta
PDA: Patent Ductus Arteriosus

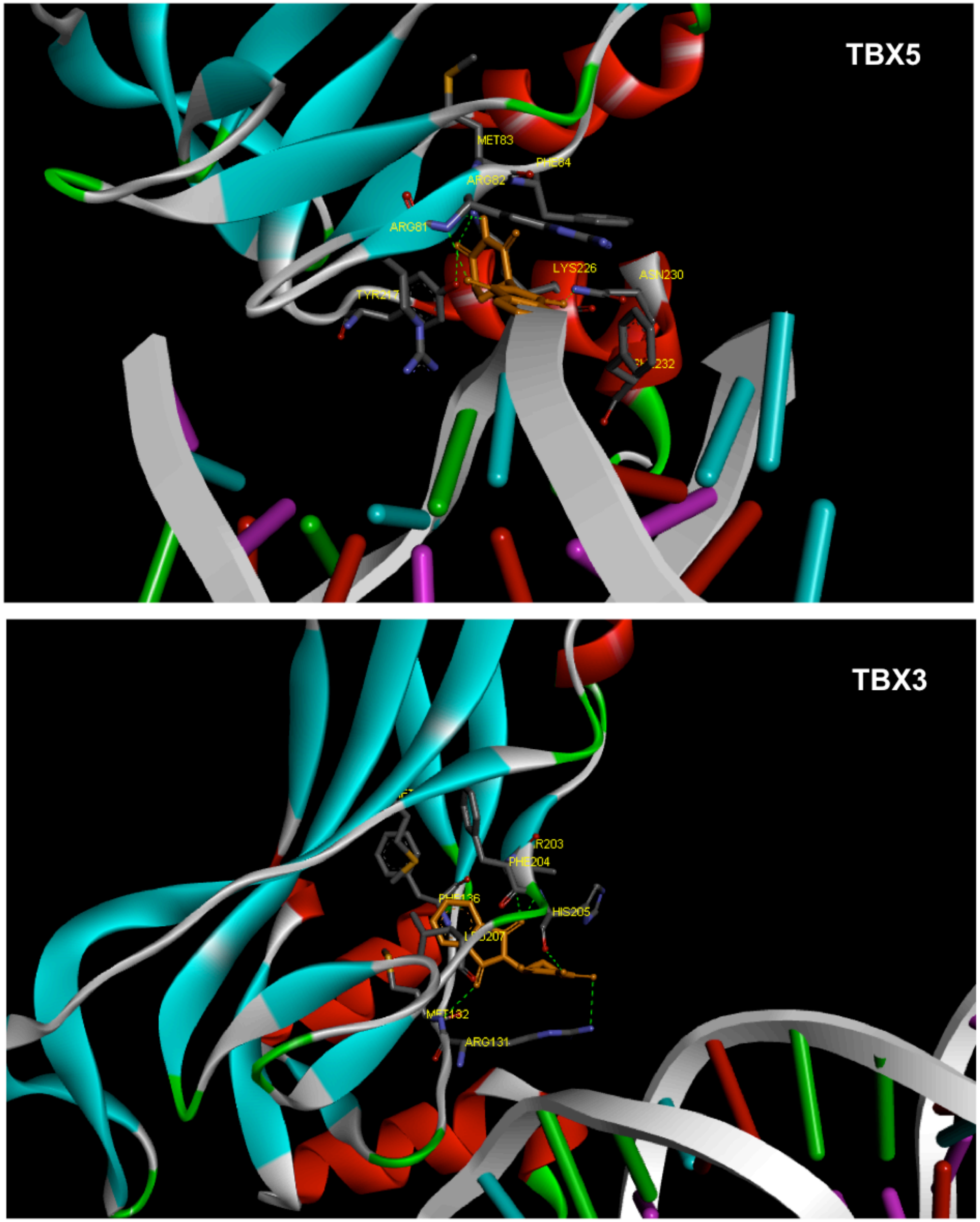

Supplementary Figure 1, Khalil A et al

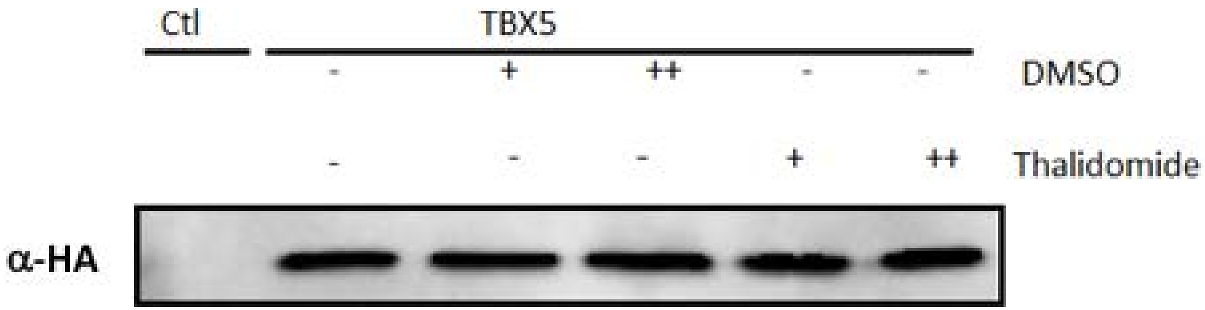

Supp Figure 2, Khalil et al

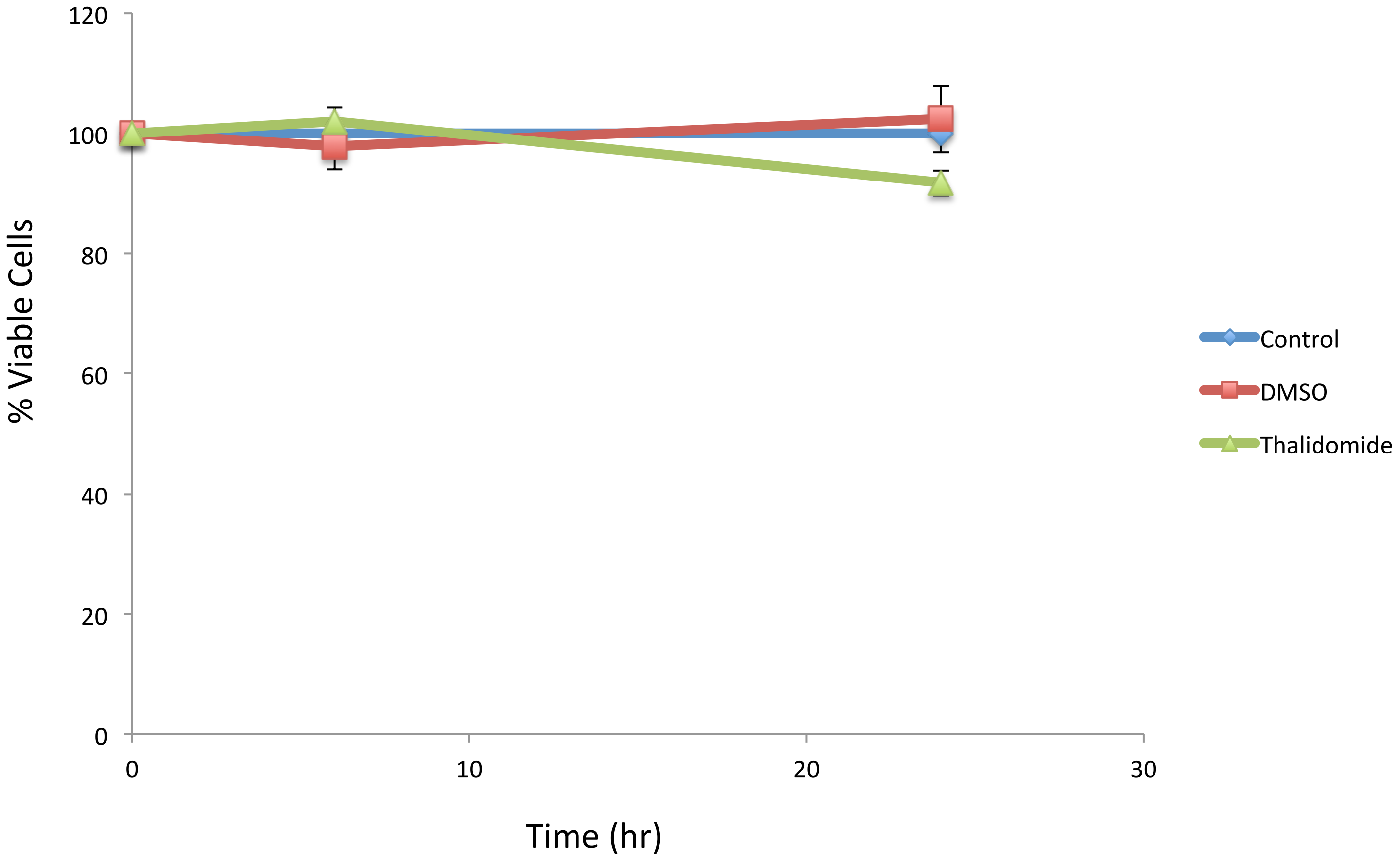

120
100
80
60
40
20
0
% Viable Cells
0
10
20
30
Time (hr)
Control
DMSO
Thalidomide

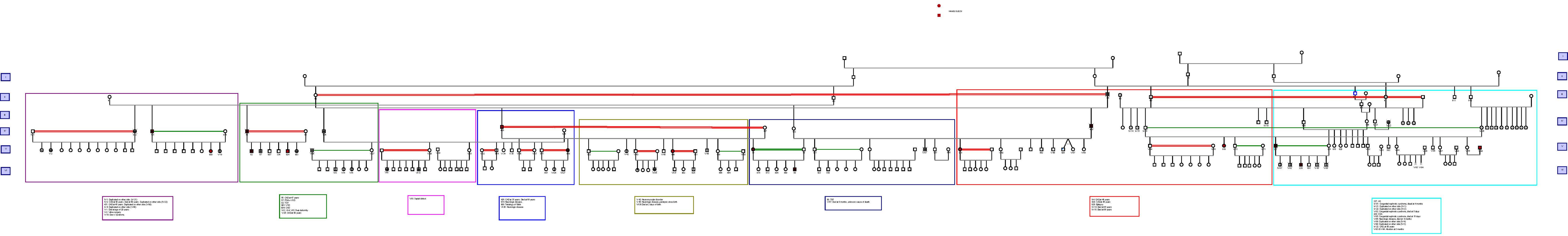

IV-1: Duplicated on other side. (IV-21)
IV-2: CAD at 50 years. Died at 60 years. Duplicated on other side (IV-22)
431: CAD at 45 years. Duplicated on other side (V-90)
IV-4: Duplicated on other side (V-89)
V-1: Died at age of 20 years
V-2: Valve surgery
V-19: Down Syndrome.
55: CAD at 47 years
57: PDA+VSD
82: TOF
903: VSD
904: CAD
VI-3, VI-4, VI-5: Feet deformity
V-26: CAD at 50 years
VI-8: Septal defect
426: CAD at 35 years. Died at 68 years
914: Neurologic disease.
908: Tetralogy of Fallot
VI-26: Neurologic disease
V-42: Neuromuscular disorder
V-48: Neurologic disease, paralysis since birth
VI-36: Died at 2 days of birth
80: TOF
V-57: Died at 8 months, unknown cause of death
II-4: CAD at 49 years
429: CAD at 45 years
926: Epilepsy
IV-14: Died at 85 years
IV-15: Died at 86 years
227: AS
IV-81: Congenital nephrotic syndrome, diead at 4 months
IV-21: Duplicated on other side (IV-1)
IV-22: Duplicated on other side (IV-2)
V-82: Congenital nephrotic syndrome, died at 5 days
229: COA
V-85: Congenital nephrotic syndrome, died at 15 days
V-86: Neurologic disease, died at 9 months
V-89: Duplicated on other side (IV-4)
V-90: Duplicated on other side (IV-3)
IV-22: CAD, at 50 years
V-93 & V-94: Abortion at 3 months
